# Heat, temperature and Clausius inequality in a model for active Brownian particles

**DOI:** 10.1038/srep46496

**Published:** 2017-04-21

**Authors:** Umberto Marini Bettolo Marconi, Andrea Puglisi, Claudio Maggi

**Affiliations:** 1 Scuola di Scienze e Tecnologie, Università di Camerino, Via Madonna delle Carceri, 62032, Camerino, INFN Perugia, Italy; 2 Consiglio Nazionale delle Ricerche-ISC, Rome, Italy; 3 NANOTEC-CNR, Institute of Nanotechnology, Soft and Living Matter Laboratory, Piazzale A. Moro 2, I-00185, Roma, Italy

## Abstract

Methods of stochastic thermodynamics and hydrodynamics are applied to a recently introduced model of active particles. The model consists of an overdamped particle subject to Gaussian coloured noise. Inspired by stochastic thermodynamics, we derive from the system’s Fokker-Planck equation the average exchanges of heat and work with the active bath and the associated entropy production. We show that a Clausius inequality holds, with the local (non-uniform) temperature of the active bath replacing the uniform temperature usually encountered in equilibrium systems. Furthermore, by restricting the dynamical space to the first velocity moments of the local distribution function we derive a hydrodynamic description where local pressure, kinetic temperature and internal heat fluxes appear and are consistent with the previous thermodynamic analysis. The procedure also shows under which conditions one obtains the unified coloured noise approximation (UCNA): such an approximation neglects the fast relaxation to the active bath and therefore yields detailed balance and zero entropy production. In the last part, by using multiple time-scale analysis, we provide a constructive method (alternative to UCNA) to determine the solution of the Kramers equation and go beyond the detailed balance condition determining negative entropy production.

Recently, there has been an upsurge of interest in active matter systems made of self-propelled particles which take energy from the environment to sustain their motion^1^,^2^. There are several reasons why this subject has drawn the attention of biologists and physicists: active particles model not only living systems such as Escherichia coli bacteria, spermatozoa, swarms of animals etc., but also manmade inanimate objects such as Janus spherical particles with catalytic patches coatings, polymeric spheres encapsulating a hematite cube, rod-shaped particles consisting of Pt and Au segments^3^ which can be studied in a laboratory. Since the constituents of active matter are powered by some external engine and constantly spend energy to move through a viscous medium, they are permanently out of equilibrium and thus provide new challenges in non equilibrium statistical mechanics^4^. Every element of an active matter system can be considered out of equilibrium, in contrast to boundary driven systems, like a system subject to a concentration gradient which is locally equilibrated.

In contrast with passive Brownian particles subject to thermal fluctuations, such as colloids in solution, active systems can be described as assemblies of particles driven by fluctuating forces which are generically correlated in time. Theoreticians have proposed several descriptions of the active dynamics including the run-and-tumble model^5^,^6^, the active Brownian particle model^7^,^8^,^9^ and the Gaussian colored noise (GCN) model^10^,^11^, where the direction of motion fluctuates, but on a short-time scale there is a persistence to move in the current directions^12^. The persistent character of their trajectories is measured experimentally through their diffusivity^3^, which is usually much larger than the diffusivity of colloidal particles. The last model is a particularly simple description of the self-propulsion mechanism, obtained by considering the motion of the particles as a set of coupled Langevin equations subject to a noise with a correlation time *τ* > 0, replacing the white noise (*τ* = 0) characterising passive matter. Thanks to this simple mathematical structure one can obtain many results in an explicit form. In particular, in the present paper we perform a study of the non equilibrium stochastic energetics of the GCN model and extend the analysis beyond the steady state and zero current regime which has been the subject of previous work of some of the authors^13^,^14^.

Stochastic energetics aims to provide a link between stochastic processes - which constitute an effective dynamical description of mesoscopic systems (i.e. where the degrees of freedom of thermostats are replaced by stochastic effective terms) - and thermodynamics^15^,^16^. It basically answers the questions of what are the heat and the work associated with a random motion and aims at tracing the role of the energy exchanges in the variation of the Gibbs entropy^17^,^18^. In macroscopic thermodynamics, one studies the change of thermodynamic entropy *S* after a transformation from the equilibrium state *A* to equilibrium state *B*, during which a heat ∫_*A*→*B*_*δQ* goes from the thermostat to the system. For such a transformation one has


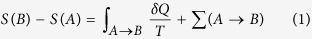


where Σ - known as entropy production - satisfies Σ ≥ 0 with the equal sign valid only in a quasi-static transformation. In a cyclic transformation *S(B*) = *S(A*), so that the Clausius inequality is recovered, 
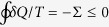
.

In stochastic thermodynamics the equilibrium entropy is replaced by Gibbs entropy *s(t*) (details are given hereafter) and the average instantaneous entropy variation is written as


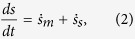


with the entropy production rate 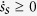 (=0 only at equilibrium) and 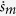 interpreted as “entropy production of the surrounding medium”^18^. Indeed in many cases of non-conservative forces applied to a system in contact with a heat bath at temperature *T*, it appears that 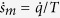, with 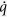 the average heat flux going from the heat bath to the system (in the rest of the paper we use, for simplicity, the notation 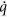 to indicate a heat flux, which of course does not imply the existence of an observable *q* depending on the state of the system). In the stationary state, therefore, one has - again -


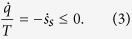


There are however other cases where such a simple interpretation of 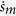 is lost. One of the most studied cases is when the non-conservative force depends on the velocity of the particle, a fact which is common in mesoscopic systems with feedback^19^ and in some models of active particle^20^,^21^,^22^,^23^. In those examples one finds a more complicate structure of the kind


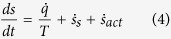


where still 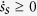, but an additional contribution 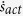 entropy production appears without a well defined sign (we use “act” for “active”, but previous authors have called it “pumping”^24^). As a consequence in the stationary state the Clausius inequality is no more guaranteed. A possible interpretation of this fact is a problem in the modellisation of the external non-conservative agent^25^.

Here we apply the methods of stochastic energetics and thermodynamics to the GCN model, showing that the instantaneous entropy variation can be written as


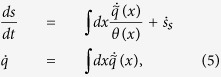


with 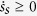 taking a simple Onsager-like structure and 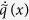 representing the local energy flux coming from the active bath (represented by the Gaussian coloured noise) which has an effective local temperature *θ(x*) (details in Section Model and Methods). Remarkably, Eq. (5) generalises the Clausius inequality^26^,^27^,^28^, as in the stationary state it implies


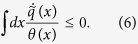


Interestingly, a known approximation of the GCN model where only the longer time-scales are considered (“UCNA” approximation^29^,^30^,^31^), yields a zero entropy production, being 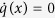 everywhere, as in local equilibrium. This is consistent with a known observation: coarse-graining operations that remove fast time-scales are likely to suppress part - or all - of the entropy production of a system^24^,^32^,^33^,^34^,^35^. For the same reason, the entropy production needed to sustain the active bath (see for instance^4^), which is usually *hotter* then the equilibrium (solvent) bath, does not appear in our description here, because the model is defined on a time-scale which is slower than the one of the molecular heat exchanges.

Equations (5, 6) are not the only important result of our work. We also derive the hydrodynamic equations of the GCN model (for non-interacting particles or, equivalently, in the dilute limit) for density, momentum, and temperature local fields. This is pivotal in getting further insight in the local thermodynamics of the model, i.e. making explicit terms such as the local pressure, local kinetic temperature, internal heat fluxes and local entropy production.

This paper is organised as follows: in section Model and Methods, we present the model of independent active particles in one dimension subject to a generic potential and write the evolution equation for the Kramers equation for the position-velocity distribution function. Using the Kramers equation and after introducing the concepts of work, heat and Gibbs entropy, we present a thermodynamic description of the non equilibrium coloured noise system by relating the entropy variation to the power and heat flux of the system. Using stochastic thermodynamic methods we show that there exists a local Clausius inequality involving the heat flux and a non uniform local temperature. Such a scenario is corroborated by the analysis of the hydrodynamic equations derived from the Kramers equation. We also derive the UCNA approximation from the hydrodynamic equations by taking the overdamped limit and show that this approximation corresponds to a vanishing entropy production which is consistent with the fact that the UCNA satisfies the detailed balance condition. To go beyond such an approximation we use a multiple-time scale method and show how a finite entropy production arises. Finally, we present our conclusions.

The Supplementary Information illustrates the multiple-time scale method used in the main text to expand the Kramers equation in powers of 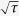 around the *τ* = 0 solution and derive the form of the phase-space probability distribution without imposing the detailed balance condition.

## Model and Methods

We consider a minimal model describing the basic dynamical properties of a suspension of mutually non-interacting active particles in the presence of an external field. The steady state properties of such a model, including interactions, have been recently studied in a series of papers, but little attention has been devoted to its dynamical properties. For the sake of simplicity we study a one dimensional model, and leave the straightforward extension to higher dimensions with interactions to future work. As mentioned in the introduction, the distinguishing feature of active matter is the ability of the self-propelled particles to convert energy from the environment into persistent motion so that one observes a variety of peculiar properties such as extraordinary large diffusivities as compared to suspensions of colloidal particles of similar size and spatial distributions not following Boltzmann statistics, to mention just a few. Besides the active Brownian particle (ABP) model, which considers independently the translational and the rotational degrees of freedom of the particles, the GCN has recently gained increasing popularity among theoreticians, because it lends itself to more analytical treatments. Farage *et al*.^11^ presented a clear discussion of how the GCN can be considered as a coarse grained version of the ABP, and can be derived by averaging over the angular degrees of freedom. The essence of the GCN is to describe the dynamics of an active suspension by means of an over damped equation for the position variable subject to a deterministic force and to a stochastic driving. At variance with colloidal solutions described by standard Brownian dynamics the noise is time-correlated to account for the persistence of the trajectories. We model the effective dynamics for the space coordinates of an assembly of non-interacting active Brownian particles^13^,^36^ as


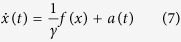


where the term *a*, also called “active bath”, evolves according to the law:


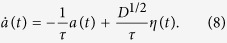


The force *f(x*) acting on each particle is time-independent and associated to the potential *w(x*), *γ* is the drag coefficient, whereas the stochastic force *η(t*) is a Gaussian and Markovian process distributed with zero mean and moments 〈*η(t)η(t*′)〉 = 2*δ(t* − *t*′). The coefficient *D* due to the activity is related to the correlation of the Ornstein-Uhlenbeck process *a(t*) via


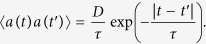


In order to proceed analytically it is convenient to switch from the (*x, a*) variables to the phase-space variables (*x, v*) where 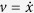 and rewrite (7) and (8) as


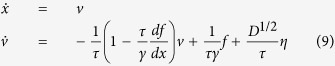


One can immediately write the associated Kramers equation for the phase-space distribution *p(x, v*;*t*):


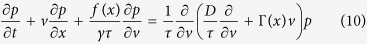


with 
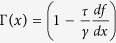
. The second and third term on the left hand side represent the streaming terms in the evolution, that is correspond to the Hamiltonian evolution of the phase-space distribution, whereas the right hand side describes the dissipative part of the evolution. Notice that, at variance with the standard Kramers equation the force is divided by the unusual factor *τγ* and the friction is space dependent and varies with *τ*.

### Transport equation in phase-space and entropy production

In order to proceed further, it is time-saving to adopt non-dimensional variables for positions, velocities, and time and rescale forces accordingly. We define 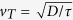 and introduce the following non-dimensional variables:


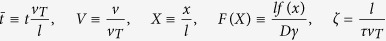


Interestingly, *ζ* is the inverse of the Péclet number, 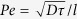, of the problem, that is the ratio between the mean square diffusive displacement due to the active bath in a time interval *τ*, the so called persistence length, over the typical length of the problem, *l*, such as length-scale of the variation of the external potential *w(x*). As it will be clear in the following the parameter *ζ* plays the role of a non-dimensional friction.

We rewrite Kramers’ evolution equation for the phase-space distribution function using (11) as:


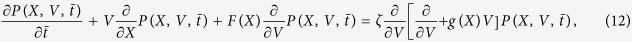


where 
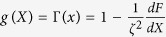
.

### Work, heat and entropy production

Equation (12) can be written as a continuity equation in phase space


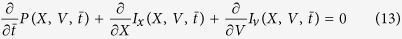


by introducing a probability current vector, (*I*_*x*_, *I*_*v*_), whose components are the sum of a reversible current (indicated with a superscript R)


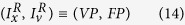


and a dissipative or irreversible current (indicated with a superscript D)


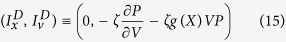


The dynamical equation (12) ruling the statistical evolution of the phase space distribution can be seen as the result of an Hamiltonian (non dissipative) dynamics coupled to an heat-bath:


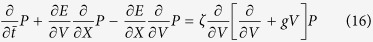


where


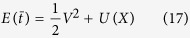


with *w* = *DγU* such that *F(X*) = −*dU*/*dX*. Upon differentiating the expectation value of *E(t*) with respect to time we obtain


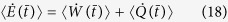


with


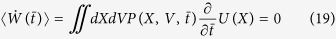



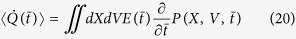



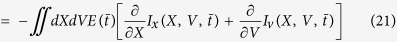



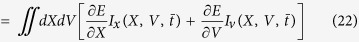


Since in the simple case of a time independent potential (which is the situation considered in the rest of this paper), 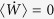, i.e. 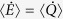. Explicitly, we obtain


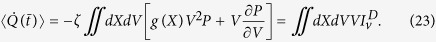


We underline that our definition of heat is coherent with the standard definition of stochastic energetics^15^. Indeed it is straightforward to verify that 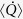 corresponds to the average of the power dissipated by forces acting on the particle which are related to the active bath, i.e. all forces appearing in Eq. (9) but the conservative term *f*/*τγ*.

We introduce now the Gibbs entropy 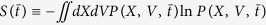, which in equilibrium systems connects the statistical level and the probability distribution 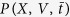 to the macroscopic thermodynamic quantities such as heat and work, and relate its time derivative to the heat rate 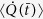. We consider:


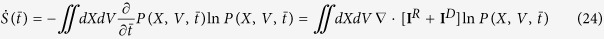


To derive equation (24) we used the continuity equation (13). After integrating by parts and using the zero flux boundary conditions at infinity and the definitions (14) and (15) we obtain the expression:


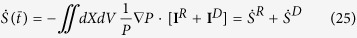



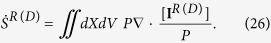


One can see that the reversible contribution to 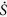 vanishes since


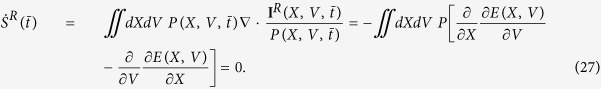


Analogously we may write the dissipative contribution to 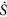 as


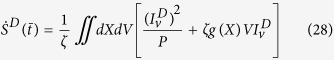


so that the total time derivative of the entropy (24) turns out to be:


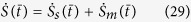



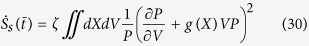



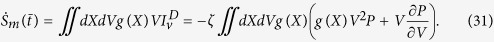


It is clear that in the steady state the time derivative of the entropy vanishes 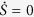, as well as 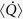. However, it is interesting to identify 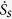 as an entropy production rate, which is indeed always non-negative, and 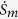 as the flux of entropy due to heat exchanges between the system and the surroundings, also known as entropy production of the medium^18^. One immediately notices that 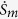 is not simply proportional to 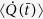, see Eq. (23), as one would find when studying the entropy production of a system coupled to an equilibrium thermostat and driven out-of-equilibrium by non-conservative forces^18^. Here we have a spatial distribution of temperatures. In order to appreciate that, we need to discuss the role of *g(X*) as an inverse temperature and to consider the dimensional form of the equations, which is done in the following two subsections.

### Absence of detailed balance condition in the GCN

The conditions of detailed balance can be written in terms of components of the dissipative or irreversible current (15), which must vanish in the steady state^37^:


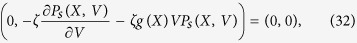


being *P*_*s*_(*X, V*) the stationary distribution. Without loss of generality, such a solution can be written as the product of a weight function *π(X, V*) and a “local” Maxwellian with velocity variance which is position-dependent:


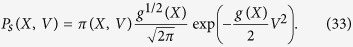


Formula (33) - inserted in Eq. (32) - requires


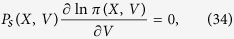


which implies that *π(X, V*) = *π(X*) is a function of positions only and can be considered as a local density.

Instead, the vanishing of the reversible part of the current vector is not a necessary condition for detailed balance, which instead requires the vanishing of its divergence. To see that, consider the case of an Hamiltonian system, where detailed balance trivially holds, but the reversible component of the current in phase space is manifestly non zero. Indeed, in the steady state in virtue of the Hamilton equations the associated divergence vanishes, thus confirming our statement. In the present case, we write the divergence of the reversible current (see Eq. (14)) as:


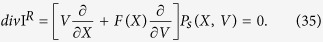


Plugging the distribution (33) into equation (35) and taking into account that the form of *P*_*s*_(*X, V*) implies the relation 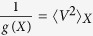, where the last average indicates the mean square value of the velocity evaluated at position *X*, we can see that a density distribution *π(X*) satisfying (35) does not exist for arbitrary choices of *g(X*). In fact, we find:


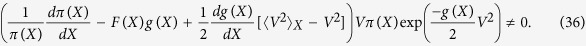


The only case where the identity holds is *g(X*) = *g*_0_, a constant, which occurs for *ζ* → ∞ which is the equilibrium limit of the model or in the cases a) of linear or b) parabolic potentials *U(X*)), and sets the equilibrium Boltzmann solution 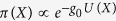 for any value of *V*. In conclusion, apart from the special case *g(X*) = *g*_0_, the Kramers eq. (12) does not satisfy the detailed balance condition. On the other hand, one may verify that the first three projections in velocity space of the zero divergence condition Eq. (36) (i.e. obtained by multiplying such an equation by (1, *V, V*^2^), respectively and integrating w.r.t. *V*) can indeed be fulfilled, and consequently, the detailed balance condition is satisfied in this velocity subspace. In the case of the multiplication by the even powers of *V* and integration by *V* the balance equation is trivially satisfied. Multiplication by *V* and integration, instead, leads to:


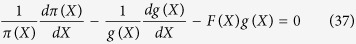


whose solution is


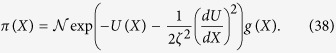


Interestingly, as we shall illustrate in below, the spatial distribution in Eq. (38) coincides with the static solution of the UCNA equation, which is known to satisfy the detailed balance condition, but in general, such a condition holds only approximately (i.e. only to second order in the parameter *ζ*^−2^) for the non equilibrium steady state under scrutiny.

### Dimensional form of equations

The dimensional form of the equations is more enlightening for a thermodynamic interpretation of the entropy production of the medium, that is the second term on the r.h.s. of Eq. (29). First of all, let us notice that the local temperature appearing in the Maxwellian Eq. (33), in dimensional form, takes the expression *θ(x*) = *D*/[*τ*Γ(*x*)]. It is also useful to define *T*_*b*_ = *D*/*τ*, so that *θ(x*) = *T*_*b*_/Γ(*x*).

The dimensional form of the total energy and of the heat flux are respectively:


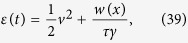


and


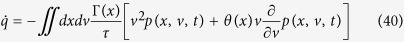


and the Gibbs entropy now reads 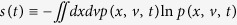. In dimensional form using the local temperature the total entropy time derivative 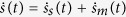 is the sum of the positive quantity


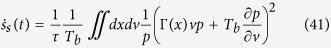


and the entropy flux


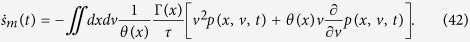


It is suggestive to rewrite


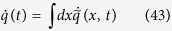



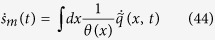


with a local density of heat flux defined as


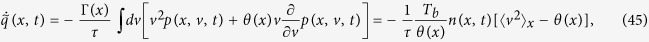


where *n(x, t*) = ∫*dvp(x, v, t*) and *n(x, t*)〈*v*^2^〉_*x*_ = ∫*dvv*^2^*p(x, v, t*), where 〈*v*^2^〉_*x*_ is the mean squared velocity at given position. Expression (44) represents an interesting connection between the local entropy production of the medium (or entropy flux) and the local heat flux divided by the same local temperature *θ(x*) = *T*_*b*_/Γ(*x*) featuring in the approximate detailed balance solution, Eq. (33). Such a result is to be compared with alternative expressions for entropy production of the medium recently derived for active systems^22^,^23^.

Remarkably, the expression (44) for the entropy production of the medium and the fact that 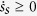, yields for the stationary state the following generalised Clausius inequality^26^


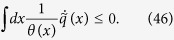


### Timescales and coarse-grained levels of description

It is interesting to discuss the characteristic timescales existing in the model and their role in the results obtained up to here. The model in Eq. (7) has its natural interpretation as a coarse-grained version of a more refined model where the particle has a mass *m*. The more refined model has three main timescales: 1) *τ*_*m*_ = *m*/*γ* which is the molecular kinetic relaxation timescale, that is the timescale of the relaxation (to the statistics of the bath) of the velocity of the particle which is achieved only when the external forces vanish, i.e. *a* = 0 and *f* = 0 (passive colloid); 2) *τ* which is the persistence timescale of the active force *a(t*); 3) *τ*_*w*_ = Δ*x*/*v*_*T*_ - where 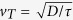 and Δ*x* is a characteristic length-scale of the potential *w(x*) - which is the time needed by the particle (roughly going at speed *v*_*T*_ which is the active bath “thermal” velocity) to see the variations of the potential *w(x*). Apart from strange choices of the potential (e.g. *w(x*) varying over very small length-scales), the natural order of the three timescales is 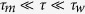. Note that *ζ* = *τ*_*w*_/*τ* (if *l* = Δ*x*) and therefore in the perturbative scheme discussed in the Supplementary Information the small parameter 1/*ζ* corresponds to 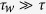.

When there is no activity (*a* = 0), one has the well-known situation of a colloid in a potential lanscape: in that case, in view of the usual separation 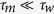 (now one uses the molecular velocity to define *τ*_*w*_ = Δ*x*/*v*_*th*_ with 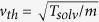 and *T*_*solv*_ is the equilibrium temperature of the solvent), one adopts the classical overdamping approach, i.e. looks at the motion of the particle on a timescale intermediate between *τ*_*m*_ and *τ*_*w*_. On such a time-scale the relaxation of the “velocity” to *f(x*)/*γ* is immediate


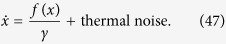


Operatively, this is equivalent to measure a “ coarse-grained velocity” defined as [*x(t* + Δ*t*) − *x(t*)]/Δ*t* with a time-lag such that 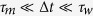. This is clearly very different from the instantaneous velocity, i.e. the one that could be measured with 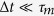, usually quite difficult in real experiments.

When there is activity *a* ≠ 0, the new timescale *τ* allows one to operate two different levels of coarse-grain. On a timescale which is intermediate between *τ*_*m*_ and *τ*, the coarse-grained velocity 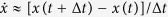 with 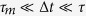 satisfies


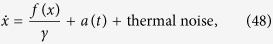


which becomes our initial model definition, Eq. (7), when the thermal noise is neglected (which is usually safe, as it is much smaller than *f(x*)/*γ* + *a(t*)).

On a much longer timescale, i.e. when the velocity is measured using 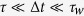, there is a complete overdamping, i.e. also the active force is averaged out and only the potential acts, but with a re-normalized viscosity *γ*Γ(*x*), i.e. the “super-coarse-grained” velocity of the particle immediately relaxes to


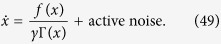


The entropy production of the first, most fundamental, level of description (real velocity, measured at 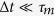) could be computed by studying its complete Fokker-Planck equation: such a small scale entropy production - however - is not particularly useful, as it would yield an expression which includes quantities which can be difficult to be measured in experiments (exceptions are presented in refs 24, 34 and 35). The meaning of this entropy production should be simple^4^: an external force (activity) is keeping the particle far from reaching thermal equilibrium (at temperature *T*_*solv*_), in particular, such a force increases the energy of the particle. There is, naturally, an energy flux from the external force (the bacterium’s engine) to the particle and a heat flux from the particle to the bath. Our paper disregards such a low-level entropy production and focuses on the entropy production at the second and third levels of description. The situation is similar to other systems where small-scale degrees of freedom are ignored (and - consequently - their contribution to entropy production), for instance in granular matter^38^.

At the second level, Eq. (48) or Eq. (7), the only relaxation of velocities which can be measured is that toward the local active bath at temperature *θ(x*) = *T*_*b*_/Γ(*x*). Its energetic counterpart is the total heat flux (Eq. (18) or - in dimensional form - Eq. (35) of the main text) which is the sum (space integral) of local heat fluxes, i.e. 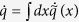. In the stationary state the total heat flux is zero: there are regions where the flux goes in one direction (e.g. the particle is hotter than *θ(x*)) compensated by regions where it goes in the opposite direction. However, even with total zero heat flux, a non-zero global entropy flux 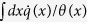 may appear: this occurs because of the non-uniformity of the temperature. In the special case of a constant Γ(*x*), *θ(x*) is proportional to *T*_*b*_ and the total entropy flux becomes proportional to the total heat flux, i.e. it vanishes. This “second-level” entropy production misses the entropy produced at the finer timescale (removed by the heat flux exchanged between the particle and the solvent heat bath), which is there even when the *θ(x*) is uniform. On the contrary, in the presence of a non-uniform *θ*, one gets a non-zero (de facto negative) total entropy flux, corresponding to positive entropy production. It is remarkable that even the incomplete description at such a second level of description yields a thermodynamic-like description where - as in the Clausius relation −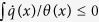. In other examples of coarse-grained out-of-equilibrium systems (e.g. granular systems^38^, but also different models of active particles^22^, or systems with feedback^19^) one has that the heat flux does not rule the entropy flux and therefore there is nothing similar to a Clausius relation^25^. Models with temperature gradients showing such a relation can be found in refs 39 and 40.

We conclude this discussion by considering the third level of description, where the relaxation of the velocity to the active heat bath is also lost, Eq. (49). This level corresponds to the UCNA approximation and its approximated velocity statistics are exactly equal, everywhere, to a local Gaussian with temperature *θ(x*) - even when it is non-uniform. Consistently with the theoretical discussion, this implies local equilibrium with the active bath, or equivalently vanishing entropy production, i.e. detailed balance.

### Internal energy and entropy density balance in dimensional form

An interesting perspective is offered by considering the properties of the hydrodynamic space only, instead of the properties of the full phase-space: this is a different kind of coarse-graining, where fast components of the full solution *p(x, v, t*) are neglected. Details of the derivation are given in the Section S1 of the Supplementary Information, where we used the non dimensional variables.

We define the local density field *n(x, t*) = ∫*dvp(x, v, t*), the local velocity field *u(x, t*) = [1/*n(x, t*)]∫*dvvp(x, v, t*), the local kinetic temperature field *T(x, t*) = [1/*n(x, t*)]∫*dvp(x, v, t*)(*v* − *u(x, t*))^2^, the local pressure *π(x, t*) = *n(x, t)T(x, t*), and the heat flux *j*_*q*_(*x, t*) = ∫*dvp(x, v, t*)(*v* − *u(x, t*))^3^/2 and get for the following hydrodynamic-like balance equations:


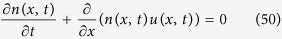



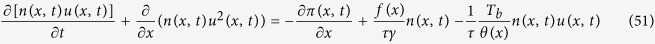



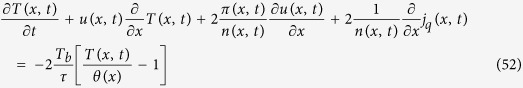


The term 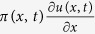 represents a compression work per unit time. The terms in the r.h.s of (51) and (52) balance equations make the approach to the local values of *u(x, t*) and *T(x, t*) fast processes, in contrast with the slow evolution of the density. Defining the internal energy as 
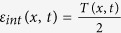
, we immediately get


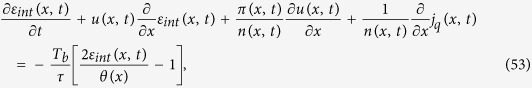


which corresponds to Eq. (34) of Chapter II in ref. 41, in the case of a system with a single chemical component. The last term, of course, is not present in ref. 41 because heat-bath thermostats are not considered there.

Using the continuity equation to eliminate the compression work in the equation for the internal energy we derive


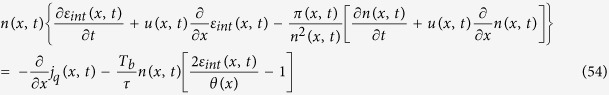


In analogy with equilibrium thermodynamics, we can identify the quantity


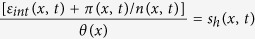


as a good candidate for the hydrodynamic entropy density and rewrite the last equation as an equation for the entropy. The first law of thermodynamics 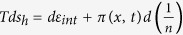 becomes the local relation


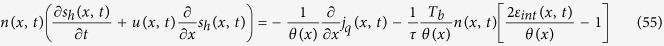


which can be also rewritten as


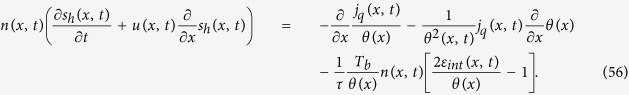


In eq. (56) we identify the *internal* entropy flux 
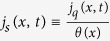
, which is the difference between the total entropy flux and the convective entropy flux^41^, and define the local entropy production of the system as:


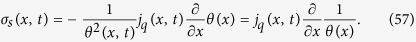


A closed expression for the heat flux *j*_*q*_(*x, t*) = *n(x, t*)〈(*v* − *u(x, t*))^3^〉/2 requires the knowledge of the third velocity moment or alternatively one must use a phenomenological closure relating heat flux to the temperature gradient, e.g. *j*_*q*_(*x, t*) = −*κ(x, t*)∇*θ(x*) with some positive thermal conductivity *κ* proportional to the density *n(x, t*). In such a case, the local entropy production, Eq. (57), is positive. Such a phenomenological assumption contrasts with the standard definition *j*_*q*_(*x, t*) = −*κ*∇*T(x, t*), however in the limit of small *τ* the temperature *T(x, t*) can be approximated by *θ(x*) so that


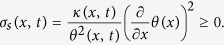


Finally, the local entropy flux towards the surrounding medium (i.e. towards the active bath) reads:


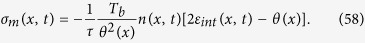


By rewriting it as


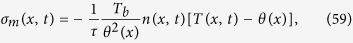


one can see that *σ*_*m*_(*x, t*) corresponds (using Eq. (45)) to the density of medium entropy production 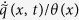 featuring in the integrand in the r.h.s. of Eq. (44).

In conclusion, we can write


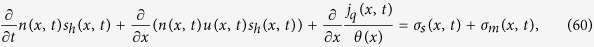


where the second and the third term on the l.h.s. together represents the entropy current density, whereas in the r.h.s. we have the total entropy production density as the sum of the entropy production of the system and of the medium. Let us remark that formula (60) is in agreement with standard treatments of non equilibrium thermodynamics^42^.

### UCNA: an approximate treatment of the Fokker-Planck-Kramers equation

In the present section, we show that it is straightforward to derive the static UCNA equation in a non perturbative fashion from the hydrodynamic eqs (50) (51) (52), 103, 104 and a series of approximations. First of all we assume that the kinetic temperature of the active particles is equal to the temperature *θ* of the non uniform heat bath: so that in eq. (52) we put


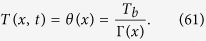


Next, we we assume that the l.h.s. of eq. (51) representing the hydrodynamic derivative of the average velocity vanishes, so that:


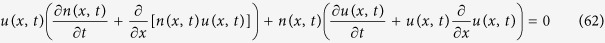


The first parenthesis vanishes by the conservation of the particle number, the second parenthesis is zero when the volume element does not accelerate, which means that there is a dynamical equilibrium between frictional forces and external forces. Thus from the momentum eq. (51) we have the balance condition:


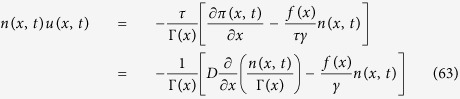


where in the last equality we have used the following relation between pressure, density and Γ(*x*):*π(x, t*) = *Dn(x, t*)/(*τ*Γ(*x*)). Notice that we have used the definition of pressure (given after Eq. (7) of the Supplementary Information):


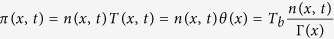


and the equality between *θ* and *T*. Such a pressure coincides with the definition of pressure in the case of the static UCNA^43^. Finally, using the continuity equation we eliminate the hydrodynamic velocity and obtain the UCNA eq.:


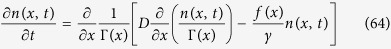


which is a modified diffusion equation. Alternatively, as shown in Section S2 of the Supplementary Information one can derive systematically by a multiple-time scale method an equation for the evolution of *n(x, t*) which is equivalent to (64) to first order in *τ*.

When the momentum current vanishes we have from (64) the following condition:


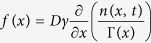


which is the hydrostatic equation discussed in previous papers by some of us^13^ showing that the dynamical and the static definitions of active pressure coincides. According to (56), the UCNA, since *j*_*q*_(*x, t*) = 0 and *T(x, t*) = *θ(x*), corresponds to


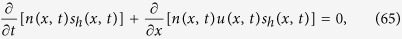


i.e. it coincides with vanishing entropy production, as previously discussed.

Finally, let us remark that due to the equality (61) the heat flux 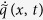 defined by (45) vanishes everywhere in the UCNA, and not only its integral. As underlined by Cates and Nardini^44^ the UCNA method maps the GCN non-equilibrium description into an equilibrium one and rules out macroscopic steady-state fluxes. Hence, *σ*_*m*_(*x, t*) vanishes within the UCNA, but we do not expect this vanishing to occur in the GCN case. In order to explicitly observe a negative value of the entropy production it is necessary to consider an approximation more refined than the UCNA, a task which will be carried out in the next section by using a perturbative method.

## Results and Discussion

### A systematic perturbative solution of the Fokker-Planck equation for the GCN

We have seen that the UCNA eq. (64), derived from the exact evolution equation (10) for the phase-space distribution function, 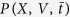 by eliminating the velocity in favour of the configurational degrees of freedom, satisfies the detailed balance condition. In Section S2 of the Supplementary Information, we show how to construct a systematic expansion of the Fokker-Planck equation in the small parameter 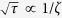, and obtain a time-dependent equation for the reduced spatial distribution function and its corrections about the solution with *τ* = 0. Such an expansion when truncated at the second order in the perturbative parameter 1/*ζ*^2^ leads to the same evolution equation for the reduced spatial density as the one introduced by Fox^45^:


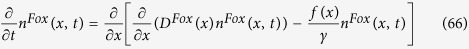


with 
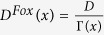
. One can observe that it has the same time independent (and zero flux) solution as the UCNA(64). Both the Fox and the UCNA description represent a contracted description in terms of a spatial distribution with respect to the phase-space distribution of the GCN, which is fully described by Kramers’ equation. The Fox approximation emerges in the limit of small *τ*, or small Péclet number, when for small *τ* the velocity distribution thermalises rapidly and reaches a gaussian shape. The method that we shall discuss below gives quantitative support to this intuitive idea. If *τ* (or Péclet) is small the particles lose memory of their initial velocities after a time span which is of the order of the time constant *τ* so that the velocity distribution soon becomes stationary. While the derivation of the UCNA equation can be done simply eliminating the time derivative of the highest order in the stochastic differential equation and then writing the associated equation for the distribution function of positions only, such a procedure does not tell us anything about the deviations of the velocity distribution from a local Maxwellian. In order to study the corrections, it is convenient to consider a general method of kinetic theory which allows extracting a reduced description from a finer one. An instance of such a method is the so-called Hilbert-Chapman-Enskog approach which allows deriving, starting from the transport equation for the phase space distribution, the Navier-Stokes equations under the form of a series expansion with the Knudsen number as the perturbation parameter (and to successive orders in Knudsen number the Burnett and super-Burnett equations)^46^. In the overdamped case, the application of the Hilbert approach is even simpler because there is only one conserved, slow mode, namely the diffusive density mode. The remaining momentum and energy variables are slaved to the density, so that one can reduce the Kramers equation to a Smoluchowski-like equation involving only the density^47^. The reduction is achieved by the multiple-time scale method as illustrated in Section S2 of the Supplementary Information where we report the necessary details of the calculation. In this section, we use some results concerning the steady state phase-space distribution function.

### Mean square velocity and entropy production beyond the order 1/*ζ* ^2^

In order to observe violations of the detailed balance condition we shall consider the fourth order in the 1/*ζ* expansion of the phase space distribution function. To this purpose, one quantity of capital interest to compute the heat-flux and the entropy production is the mean square velocity. Using the expansion of the steady state *P(X, V*) given in Eq. (28) of the Supplementary Information up to fourth order in *ζ*^−1^ we have the formula for 〈*V*^2^〉_*X*_, the dimensionless mean squared velocity at given position, which is also the local kinetic temperature of the particle:


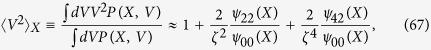


where *ψ*_00_(*X*) = ∫*dVP(X, V*) is the marginalized distribution function and ∫*dVV*^2^*P(X, V*) = *ψ*_00_(*X*) + 2*ψ*_22_(*X*)/*ζ*^2^ + 2*ψ*_42_(*X*)/*ζ*^4^ + *O(ζ*^−6^) according to Eq. (28) of the Supplementary Information. Substituting eqs (18) and (25) from the Supplementary Information we obtain the following approximation:


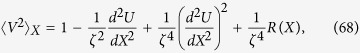


where the remainder, *R(X*) is found using the result of Eq. (25) in the Supplementary Information:


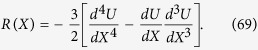


We compare now the expression (68) with the approximate UCNA prediction:


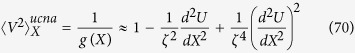


and conclude that up to order *ζ*^−2^ the result (68) of the multiple-time scale method and of the UCNA agree and give the same value the kinetic temperature 〈*V*^2^〉_*X*_. On the other hand, at order *ζ*^−4^ the two formulae are identical only when *R(X*) = 0, a situation occurring both in the case of a particle confined to an harmonic potential well or in a constant force field, where the entropy production vanishes, in agreement with the fact that the detailed balance holds.

Let us consider the difference, Δ(*X*), between the local temperature of the bath 1/*g(X*) and the kinetic temperature 〈*V*^2^(*X*)〉_*X*_ whose sign controls the direction of the local heat exchange with the bath (positive values correspond to an heat flux towards the particle, whereas for negative values the particle transfers heat to the bath):


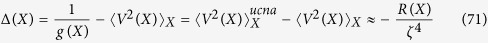


where the last approximate equality follows from (68). Thus inserting this result in eq. (31) we find:


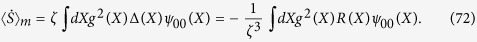


Using the scaling 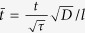 we can see that the lowest order estimate of the dimensional entropy production rate 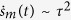 in agreement with Fodor *et al*.^48^.

### A numerical example

**Figure 1:**
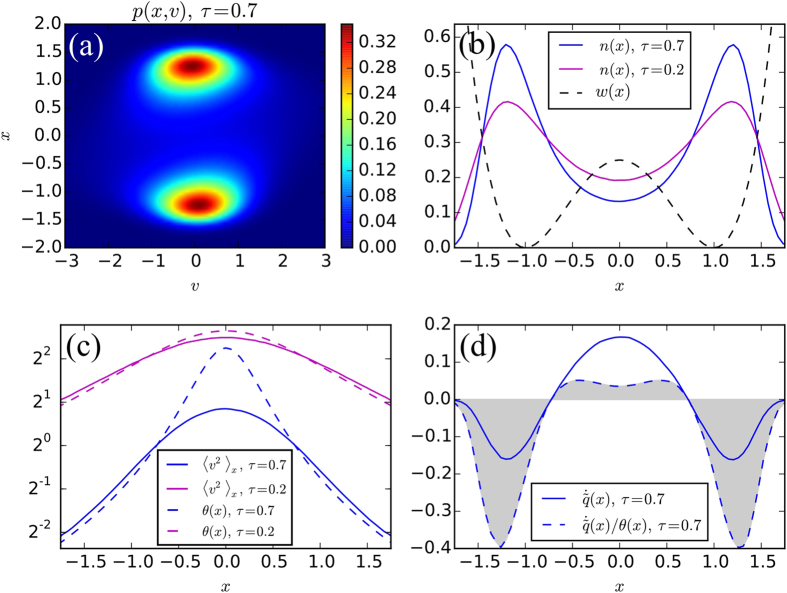
(**a**) Stationary probability distribution *p(x, v*), obtained numerically in the case of a double-well potential and persistence time *τ* = 0.7. (**b**) Position probability distribution *n(x*) (full line) obtained numerically for the potential *w(x*) (dashed line) for two different values of the persistence time (blue *τ* = 0.7 and magenta *τ* = 0.2). The potential is shifted upwards by an inessential constant 1/4 for reasons of presentation. (**c**) “Temperature” profiles 〈*v*^2^〉_*x*_ and *θ(x*) (full and dashed lines respectively and blue *τ* = 0.7 and magenta *τ* = 0.2). In the case *τ* = 0.7, notice the crossover of the difference *θ(x*) − 〈*v*^2^〉_*x*_ from positive values at small values of *x* to negative values at larger values of the coordinate. When *τ* = 0.2, the difference is very small. (**d**) Local heat flow and local entropy production (full and dashed lines respectively) for persistence time *τ* = 0.7. Both quantities are negative in the potential wells since there the particle transfers heat to the bath, whereas the opposite occurs in the peak region. Note that the integral of the entropy is negative as evidenced by the grey area, in agreement with (46).

In order to illustrate the above theoretical results, we have performed some numerical simulations and integrated the stochastic eq. (9) by an Euler algorithm optimized for GPU execution^36^. We focus on the archetypical case of the double well potential: *w(x*) = *x*^4^/4 − *x*^2^/2. This potential has been used as the simplest model for testing the colored noise approximation schemes^29^ and it has been recently considered as a Ginzburg-Landau potential for the phase separation of attractive active particles^49^. For this example we set *γ* = 1, *D* = 1 and *τ* = 0.7, we use a time-step *dt* = 10^−3^*τ* and we average 1024 independent trajectories staring in *x* = 0 for more than 10^7^ steps. In Fig. 1(a) we report the full phase-space distribution *p(x, v*), the corresponding *p(x*) is shown in Fig. 1(b). In Fig. 1(c) we show the “temperature” profiles 〈*v*^2^〉_*x*_ and *θ(x*) determining the local heat flow 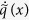 (equation (45)) and the local entropy production 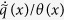 (see (44)) shown in Fig. 1(d). We find in agreement with the theory of section Model and Methods that in the stationary state 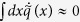 as shown in Fig. 1(d) the positive lobe of 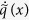 is compensated by two smaller negative lobes. Differently for the entropy we have 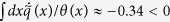 in agreement with inequality (46) as also shown in Fig. 1(d) where 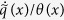 has much more pronounced negative lobes (dashed line, gray area). Let us remark that these numerical findings are in agreement with our theoretical formula (74) for the velocity difference.

Finally, we can compare these results with the prediction of the theory of the previous subsection, use the quartic potential *X*^4^/4 − *X*^2^/2 and employ (69) to evaluate the mean square velocity


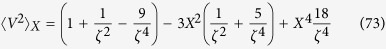


and observe that in this case 〈*V*^2^〉_*X*_ differs from 1/*g(X*) (the non dimensional equivalent of *θ(x*)) by a quantity −Δ(*X*), with:


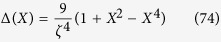


which assumes positive values near the origin, but is negative for large values of *X* as also shown by the numerical solution Fig. 1(c). The negativity of Δ(*X*) for large *X* can explain why the entropy production predicted by eq. (72) is negative in agreement with the numerical result of Fig. 1(d).

## Summary and Conclusion

We have analysed the energetics and thermodynamics of a stochastic model for active particles, in the non-interacting case and restricted to a single dimension. Our results show that the active bath (the fluctuating active force *a(t*)) can be interpreted as at local equilibrium at temperature *θ(x*) = *T*_*b*_/Γ(*x*) = *D*/[*τ*Γ(*x*)], with *D* the active diffusivity, *τ* the active persistence and Γ(*x*) a renormalisation factor which depends on the external force field. As a matter of fact, the particle is at equilibrium with the active bath (yielding a zero entropy production) only when the force field is flat, i.e. Γ(*x*) = 1. Otherwise, there is an active entropy production which - in the stationary state - is eliminated as entropy flux to the medium, taking the simple expression Eq. (44) and obeying a generalised Clausius relation, Eq. (46). By observing the system on a longer timescale (UCNA approximation), the discrepancy between local temperature and *θ(x*) becomes negligible and the particle appears as at equilibrium with the active bath. A hydrodynamic approach which describes the evolution of density, velocity and temperature fields, allows one to define local internal energy, local entropy and their local “thermodynamic” relations. The result is consistent with the global picture obtained from the analysis of the Kramers equation. In the hydrodynamic description, the UCNA approximation is interpreted as the analog of the Euler hydrodynamic solution of the Boltzmann equation. There one employs a local Maxwell-Boltzmann distribution and obtains an approximate solution of the transport equation and a set of conditions which determine the local values of the density, fluid velocity and temperature, the hydrodynamic fields. In the present case, the solution of the Kramers equation is a local Maxwellian at temperature 1/*g(X*) and density *π(X*) given by the UCNA. Finally, we have discussed how to improve the theory and obtain a non vanishing entropy production by deriving a systematic expansion of the phase-space distribution function in powers of 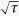 without invoking the detailed balance condition. When this condition is violated we observe numerically and theoretically a negative entropy production and a dependence of the local mean square velocity on position.

As far as approximations, such as the UCNA, involving the detailed balance condition, are concerned their predictions about the steady state structure of many-particle systems should be valid up to order 1/*ζ*^2^, i.e. order *τ*. In this case, the application of the concept of effective potential can lead to simple treatments of non uniform systems but are not reliable when the activity becomes large and higher order terms in the perturbative expansion are important.

A generalisation of the above discussion to many dimensions and to interacting particles is expected to give similar results, but certainly, deserves further investigation. A promising line of research is an experimental verification of the generalised Clausius inequality, Eq. (46).

## Additional Information

**How to cite this article:** Marconi, U. M. B. *et al*. Heat, temperature and Clausius inequality in a model for active Brownian particles. *Sci. Rep.* **7**, 46496; doi: 10.1038/srep46496 (2017).

**Publisher's note:** Springer Nature remains neutral with regard to jurisdictional claims in published maps and institutional affiliations.

## Supplementary Material

Supplementary Information
