# Erratum: Heat, temperature and Clausius inequality in a model for active Brownian particles

**DOI:** 10.1038/srep46870

**Published:** 2018-06-08

**Authors:** Umberto Marini Bettolo Marconi, Andrea Puglisi, Claudio Maggi

Scientific Reports 7: Article number: 4649610.1038/srep46496; published online: 04 21 2017; updated: 06 08 2018

The original version of this Article contained an error in the Accepted date ‘17 March 2017’ which was incorrectly given as ‘17 March 2015’. This error has now been corrected in the PDF and HTML versions of the Article.

