# Supplementary material for: Heat, temperature and Clausius inequality in a model for active Brownian particles
Source: Sci Rep. 2017 Apr 21;7:46496. doi: 10.1038/srep46496 (PMC5399351; doi:10.1038/srep46496)
Supplement: Supplementary Information [file srep46496-s1.pdf]

Heat, temperature and Clausius inequality in a model for active Brownian particles
SUPPLEMENTARY INFORMATION

Umberto Marini Bettolo Marconi
Scuola di Scienze e Tecnologie, Università di Camerino,
Via Madonna delle Carceri, 62032, Camerino, INFN Perugia, Italy

Andrea Puglisi
Consiglio Nazionale delle Ricerche-ISC, Rome, Italy

Claudio Maggi
NANOTEC-CNR, Institute of Nanotechnology, Soft and Living Matter Laboratory, Piazzale A. Moro 2, I-00185, Roma, Italy

S1. ENERGETICS OF THE HYDRODYNAMIC EQUATIONS

A well-known procedure to derive from the transport equation the coupled evolution equations for the density, the momentum density, and the average kinetic energy is to project the the transport equation over the subspace $(1, V, V^2)$, that is multiply Eq. (12) of the main text by these functions and integrate over V. This method leads to the first three members of an infinite hierarchy of equations for the velocity moments. Such equations are nothing else as the balance equations for number density, momentum density, and kinetic energy density.

$$\frac{\partial \rho(X,\bar{t})}{\partial \bar{t}} + \frac{\partial}{\partial X} J_v(X,\bar{t}) = 0 \tag{1}$$

$$\frac{\partial J_v(X,\bar{t})}{\partial \bar{t}} + 2\frac{\partial K(X,t)}{\partial X} - F(X)\rho(X,\bar{t}) = -\zeta g(X) J_v(X,\bar{t}) \tag{2}$$

$$\frac{\partial K(X,\bar{t})}{\partial \bar{t}} + \frac{\partial J_k(X,t)}{\partial X} - F(X) J_v(X,\bar{t}) = -2\zeta g(X) K(X,\bar{t}) + \zeta \rho(X,\bar{t})\,, \tag{3}$$

where we have introduced the number density $\rho(X,\bar{t}) = \int dV P(X,V,\bar{t})$, the momentum density $J_v(X,\bar{t}) = \int dV V P(X,V,\bar{t})$, the kinetic energy density $K(X,\bar{t}) = 1/2 \int dV V^2 P(X,V,\tau)$ and kinetic energy flux $J_k(X,\bar{t}) = 1/2 \int dV V^3 P(X,V,\bar{t})$. Notice that the presence of the last term in eq. (3) tends to maintain the fluid at local temperature $1/g(X)$ (which in dimensional form corresponds to the temperature $\theta(x)$.

If the hierarchy of moment equations is truncated, by supplementing the constitutive equations, one recovers the analog of hydrodynamic equations with dissipation.

Let us integrate Eqs. (2) and (3) over whole phase space (so that the integral of spatial gradients vanish). We get

$$\frac{d}{d\bar{t}} \int dX J_v(X,\bar{t}) = \int dX \Big[F(X)\rho(X,\bar{t}) - \zeta g(X) J_v(X,\bar{t})\Big] \tag{4}$$

$$\frac{d}{d\bar{t}} \int dX K(X,\bar{t}) = \int dX \Big[F(X) J_v(X,\bar{t}) - 2\zeta g(X) K(X,\bar{t}) + \zeta \rho(X,\bar{t})\Big] \tag{5}$$

If the particle (described by the current J_v) does not accelerate the l.h.s. of eq. (4) vanishes:

$$\int dX F(X)\rho(X,\bar{t}) = \zeta \int dX g(X) J_v(X,\bar{t}), \tag{6}$$

and if the space averaged kinetic energy, represented by the l.h.s.of (5) does not change, the work done per unit time by the external force (power) equals the frictional dissipation

$$\int dX F(X) J_v(X,\bar{t}) = \zeta \int dX \Big[2g(X)K(X,\bar{t}) - \rho(X,\bar{t})\Big]. \tag{7}$$

S2. MULTIPLE TIME-SCALE ANALYSIS OF THE KRAMERS EQUATION

In this Section, we derive the Fox equation for the distribution function [1, 2] by a multiple time-scale analysis following the same method employed in ref. [3, 4]. It allows deriving in a systematic fashion the configurational Smoluchowski equation from the Kramers equation via the elimination of the velocity degrees of freedom (the multiple time scale method presented here is different from the one we recently reported because to perform the perturbative calculation we employ an Hermite uniform basis in this Section [5]).

In order to perform the multiple-time scale according to the method of Titulaer [6], we rewrite Eq. (12) of the main text using the operator $L_{FP} = \frac{\partial}{\partial V}\Big[\frac{\partial}{\partial V} + V\Big]$:

$$\frac{\partial P(X,V,\bar{t})}{\partial \bar{t}} + V\frac{\partial}{\partial X}P(X,V,\bar{t}) + F(X,\bar{t})\frac{\partial}{\partial V}P(X,V,\bar{t}) + \frac{1}{\zeta}\frac{\partial F}{\partial X}\frac{\partial}{\partial V} V P(X,V,\bar{t}) = \zeta L_{FP} P(X,V,\bar{t}). \tag{8}$$

The idea, is to use the non-dimensional parameter ζ^{-1}, which vanishes when $\tau \to 0$ at fixed v_T, to perform a perturbative expansion by projecting eq. (8) onto the Hermite eigenfunctions $H_\nu(V) = (-1)^\nu \frac{1}{\sqrt{2\pi}} \frac{\partial^\nu}{\partial V^\nu} \exp(-\frac{1}{2}V^2)$. of

of L_{FP} corresponding to the eigenvalues $\nu = 0, -1, -2, .., -\nu$. Since the Hermite basis is complete and orthogonal, one may represent the solution $P(X, V, \bar{t})$ as a linear superposition of $H_\nu(V)$ with time and space dependent coefficients $\phi_\nu(X, \bar{t})$

$$P(X, V, \bar{t}) \equiv \sum_{\nu=0}^{\infty} \phi_\nu(X, \bar{t}) H_\nu(V). \tag{9}$$

We shall prove that to leading order in ζ^{-1} the time-dependent coefficients in (9) with $\nu > 0$ vanish as $e^{-\nu\zeta\bar{t}}$, accounting for the fast relaxation of the momentum, kinetic energy and higher velocity moments of the distribution P, whereas the mode with $\nu = 0$ displays the slowest decay and after a transient of the order ζ^{-1} will provide the largest contribution to the series (9) . The machinery to construct such a solution employs slow and fast time-scale variables, $\bar{t}_0, \bar{t}_1, \bar{t}_2, ...$ replacing the original the time variable, $\bar{t}$. They are related to the original variable by $\bar{t}_n = \zeta^{-n}\bar{t}$ and treated as if they were independent. Thus, the physical time-dependent function, $P(X, V, \bar{t})$, is replaced by an auxiliary function, $P_a(X, V, \bar{t}_0, \bar{t}_1, ..)$, that depends on all $\bar{t}_n$. By using a perturbation theory in powers of ζ^{-1} illustrated below one constructs order by order the solution, i.e. determines the coefficients of the series (9) in terms of the coefficients of lower order in ζ^{-1} and once the coefficients corresponding to the various orders have been determined, one returns to the original time variable and to the original distribution. One begins by replacing the time derivative with respect to $\bar{t}$ by a sum of partial derivatives:

$$\frac{\partial}{\partial \bar{t}} = \frac{\partial}{\partial \bar{t}_0} + \frac{1}{\zeta}\frac{\partial}{\partial \bar{t}_1} + \frac{1}{\zeta^2}\frac{\partial}{\partial \bar{t}_2} + .. \tag{10}$$

and expands the coefficients ϕ_ν in powers of ζ^{-1}

$$P_a(X, V, \bar{t}_0, \bar{t}_1, \bar{t}_2, ..) = \sum_{s=0}^{\infty} \frac{1}{\zeta^s} \sum_{\nu=0}^{\infty} \psi_{s\nu}(X, \bar{t}_0, \bar{t}_1, \bar{t}_2, ..) H_\nu(V). \tag{11}$$

One, now, substitutes the time derivative (10) and expression (11) into eq. (8) and equates terms of the same order in ζ^{-1}. This procedure gives a hierarchy of relations between the amplitudes $\psi_{s\nu}$, allowing to express those of order $s > 0$ in terms of ψ_{00}. To order ζ^0 eq. (8) gives:

$$L_{FP}\Big[\sum_\nu \psi_{0\nu} H_\nu\Big] = 0 \tag{12}$$

which shows that only the amplitude ψ_{00}, associated with the null eigenvalue ($\nu = 0$), is non vanishing. Next, we consider terms of order ζ^{-1} and obtain:

$$L_{FP}\sum_{\nu>0}\Big[\psi_{1\nu} H_\nu\Big] = \frac{\partial \psi_{00}}{\partial \bar{t}_0} H_0 + \Big(V\frac{\partial}{\partial X} + F\frac{\partial}{\partial V}\Big) H_0 \psi_{00} \tag{13}$$

After some straightforward calculations and equating the coefficients multiplying the same Hermite polynomial we find:

$$\frac{\partial \psi_{00}}{\partial \bar{t}_0} = 0 \tag{14}$$

$\psi_{1\nu} = 0$ when $\nu > 1$ and

$$\psi_{11} = -D_X \psi_{00} \tag{15}$$

with $D_X \equiv \left(\frac{\partial}{\partial X} - F\right)$. According to (14) the amplitude ψ_{00} is constant with respect to $\bar{t}_0$ and so is ψ_{11} being a functional of ψ_{00}. The equations of order ζ^{-2} give the following conditions:

$$\frac{\partial \psi_{00}}{\partial \bar{t}_1} = -\frac{\partial}{\partial X}\psi_{11} = \frac{\partial}{\partial X} D_X \psi_{00} \tag{16}$$

$$\psi_{2\nu} = 0 \quad \nu \neq 2 \tag{17}$$

$$\psi_{22} = -\frac{1}{2}[D_X \psi_{11} - \frac{dF}{dX}\psi_{00}] = \frac{1}{2}\frac{dF}{dX}\psi_{00} \tag{18}$$

The third order order, ζ^{-3}, equations give:

$$\frac{\partial\psi_{00}}{\partial\bar{t}_2} = 0 \tag{19}$$

$$\psi_{31} = -\frac{\partial}{\partial X}[\frac{dF}{dX}\psi_{00}] = -[F\frac{dF}{dX} + \frac{d^2F}{dX^2}]\psi_{00} \tag{20}$$

$$\psi_{32} = 0 \tag{21}$$

$$\psi_{33} = -\frac{1}{3}[D_X\psi_{22} - \frac{dF}{dX}\psi_{11}] = -\frac{1}{6}\frac{d^2F}{dX^2}\psi_{00} \tag{22}$$

$$\tag{23}$$

Finally the order ζ^{-4} of the expansion yields

$$\frac{\partial\psi_{00}}{\partial\bar{t}_3} = -\frac{\partial}{\partial X}\psi_{31} = \frac{\partial^2}{\partial X^2}[\frac{dF}{dX}\psi_{00}] \tag{24}$$

and

$$\psi_{42} = -\frac{1}{2}D_X\psi_{31} - \frac{3}{2}\frac{\partial}{\partial X}\psi_{33} = \frac{1}{4}\psi_{00}\Big[2(\frac{dF}{dX})^2 + 3\frac{d^3F}{dX^3} + 3F\frac{d^2F}{dX^2}\Big] \tag{25}$$

Putting together results (15) and (20) and restoring the original time variable $\bar{t}$ we obtain at the following closed equation for ψ_{00}:

$$\frac{\partial\psi_{00}(X,\bar{t})}{\partial\bar{t}} = \frac{1}{\zeta}\frac{\partial}{\partial X}\Big[\Big(\frac{\partial}{\partial X} - F\Big)\psi_{00} + \frac{1}{\zeta^2}\frac{\partial}{\partial X}\Big(\frac{dF}{dX}\psi_{00}\Big)\Big] \tag{26}$$

We, now, compare such a result with Fox's result at the same order in the perturbation parameter: let us write Fox equation (Eq. (66) of the main text) in non dimensional form as

$$\frac{\partial\psi_{00}^{Fox}(X,\bar{t})}{\partial\bar{t}} = \frac{1}{\zeta}\frac{\partial}{\partial X}\Big[\frac{\partial}{\partial X}\frac{\psi_{00}^{Fox}}{1-\frac{1}{\zeta^2}\frac{dF}{dX}} - F\psi_{00}^{Fox}\Big] \approx \frac{1}{\zeta}\frac{\partial}{\partial X}\Big[\Big(\frac{\partial}{\partial X} - F\Big)\psi_{00}^{Fox} + \frac{1}{\zeta^2}\frac{\partial}{\partial X}\Big(\frac{dF}{dX}\psi_{00}^{Fox}\Big)\Big] \tag{27}$$

where the last approximate equality follows from taking the large ζ limit (i.e. small τ) and reproduces the result of the multiple time scale expansion. Solving eq. (27) in the steady state we obtain:

$$\psi_{00}(X) \approx A\exp\Big(-U(X) - \frac{1}{2\zeta^2}(\frac{dU}{dX})^2\Big)(1 + \frac{1}{\zeta^2}\frac{d^2U}{dX^2}).$$

Moreover, we have found that the phase space distribution, even when the momentum current vanishes, contains terms proportional to $\bar{H}_2(V), \bar{H}_3(V)$... thus showing the non equilibrium nature of the state. To see that, let us write

$$P(X,V,\bar{t}) \equiv \sum_{\nu=0}^{\infty}\phi_\nu(X,\bar{t})H_\nu(V)$$
$$= \psi_{00}(X,\bar{t})H_0(V) + [\frac{1}{\zeta}\psi_{11}(X,\bar{t}) + \frac{1}{\zeta^3}\psi_{31}(X,\bar{t})]H_1(V) + \frac{1}{\zeta^2}\psi_{22}(X,\bar{t})H_2(V) + \frac{1}{\zeta^3}\psi_{33}(X,\bar{t})H_3(V) + \dots \tag{28}$$

Thus, the multiple-time scale method gives information about the moments with $\nu > 0$ which are not considered in UCNA and in Fox's theories since these only deal with the configurational part of the distribution function. The coefficients $\psi_{11}, \psi_{31}, \psi_{22}, \psi_{33}$ can be computed from eqs. (15), (20),(22). Notice that even in the steady state, the velocity cumulants of order $\nu \geq 2$ do not vanish, showing that the solution does not converge to a Maxwell-Boltzmann distribution corresponding to $\psi_{00} \neq 0$ and to the vanishing of all remaining coefficients $\psi_{\nu k}$.

[1] Fox, R. F. Functional-calculus approach to stochastic differential equations. *Physical Review A* **33**, 467 (1986).

[2] Fodor, É. *et al.* How far from equilibrium is active matter? *arXiv preprint arXiv:1604.00953* (2016).

[3] Marconi, U. M. B. & Tarazona, P. Nonequilibrium inertial dynamics of colloidal systems. *The Journal of Chemical Physics* **124**, 164901 (2006).

[4] Marini Bettolo Marconi, U., Tarazona, P. & Cecconi, F. Theory of thermostatted inhomogeneous granular fluids: A self-consistent density functional description. *The Journal of Chemical Physics* **126**, 164904 (2007).

[5] Marconi, U. M. B., Paoluzzi, M. & Maggi, C. Effective potential method for active particles. *Molecular Physics* 1–11 (2016).

[6] Titulaer, U. M. A systematic solution procedure for the fokker-planck equation of a brownian particle in the high-friction case. *Physica A: Statistical Mechanics and its Applications* **91**, 321–344 (1978).
